# White Matter Tract Alterations in Bipolar Disorder: A Systematic Review and Meta‐Analysis of Diffusion Tensor Imaging Studies

**DOI:** 10.1002/brb3.71459

**Published:** 2026-04-30

**Authors:** Behnaz Mahmoudvand, Mohammad Saeed Soleimani Meigoli, Alisa Mohebbi, Negarsadat Namazi, Mina Jafari, Mohammad Sadra Saghafi, Milad Yousefi, Mahsa Asadi Anar, Amirhossein Rigi, Seyed Amirali Zakavi, Mohammad Javad Khosravi, Farbod Khosravi, Zahra Rahimian, Hadi Mahdavian, Hanna Torkzadeh, Melika Arab Bafrani, Shaghayegh Karami, Shadi Hajizamani, Mohaddeseh Sadat Hosseini

**Affiliations:** ^1^ School of Medicine Iran University of Medical Sciences Tehran Iran; ^2^ School of Medicine Fasa University of Medical Sciences Fars Iran; ^3^ School of Medicine Tehran University of Medical Sciences Tehran Iran; ^4^ School of Medicine Shahid Beheshti University of Medical Sciences Tehran Iran; ^5^ School of Medicine Golestan University of Medical Sciences Gorgan Iran; ^6^ School of Medicine Qom University of Medical Sciences Qom Iran; ^7^ Institute for Cognitive and Brain Sciences Shahid Beheshti University Tehran Iran; ^8^ Department of Radiology Shahid Beheshti University of Medical Sciences Tehran Iran; ^9^ School of Medicine Ardabil University of Medical Sciences Ardabil Iran; ^10^ School of Medicine Zahedan University of Medical Sciences Zahedan Iran; ^11^ School of Medicine Shiraz University of Medical Science Shiraz Iran; ^12^ Department of Psychology Shahid Bahonar University of Kerman Kerman Iran; ^13^ Department of Neuroscience, School of Advanced Medical Sciences and Technologies Shiraz University of Medical Sciences Shiraz Iran

**Keywords:** bipolar disorder, diffusion tensor imaging (DTI), exploratory spatial clustering, fractional anisotropy, MNI coordinate analysis, neuroimaging biomarkers, white matter

## Abstract

**Background:**

White matter microstructural changes have been frequently reported in bipolar disorder (BD), yet the magnitude and anatomical consistency of these alterations remain unclear across studies.

**Methods:**

A systematic search of PubMed, Web of Science, and Scopus identified diffusion tensor imaging (DTI) studies comparing fractional anisotropy (FA) between individuals with BD and healthy controls. After screening, 145 studies were included in the systematic review. Of these, 28 studies provided tract‐level FA data suitable for quantitative synthesis, yielding 78 independent region‐specific effect‐size entries, analyzed using random‐effects models. Participant counts were deduplicated at the study level.

**Results:**

Across the 145 studies included in the systematic review, there were 5372 participants with BD and 6240 healthy controls. Within the 28‐study quantitative synthesis, pooled analyses suggested lower FA in BD across several white matter tracts, particularly those involved in fronto‐limbic regulation and interhemispheric integration. Effect sizes and heterogeneity varied by tract, reflecting methodological, demographic, and clinical diversity among the quantitatively synthesized studies.

**Conclusions:**

These findings support the presence of region‐specific white matter alterations in BD, particularly within tracts associated with emotional regulation, cognitive control, and hemispheric communication. While the results add anatomical detail to the existing evidence base, further harmonized, longitudinal, and multimodal studies are needed to examine clinical specificity, developmental timing, and biomarker potential.

## Graphical abstract

This systematic review and meta‐analysis synthesizes diffusion tensor imaging evidence of white matter microstructural abnormalities in bipolar disorder. Across published studies, patients with bipolar disorder showed reduced fractional anisotropy in several major white matter tracts, including the corpus callosum, cingulum bundle, corona radiata, anterior thalamic radiation, uncinate fasciculus, superior longitudinal fasciculus, corticospinal tract, and external capsule. Spatial synthesis of reported coordinates and voxel extent further suggested recurring involvement of interhemispheric, fronto‐limbic, and projection pathways. Together, these findings support the presence of distributed but anatomically selective white matter disruption in bipolar disorder and highlight the potential value of tract‐level diffusion markers within future multimodal biomarker frameworks.

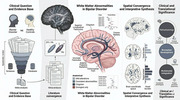

## 1 Introduction

Bipolar disorder (BD) is a chronic and debilitating psychiatric illness characterized by recurrent episodes of mania, hypomania, and depression (Barbuti et al. 2023). These episodic mood disturbances result in significant functional impairment, poor quality of life, and increased mortality due to suicide and comorbid medical conditions. BD is commonly categorized into bipolar I disorder (BD‐I) and bipolar II disorder (BD‐II), with lifetime prevalence rates estimated to be between 1% and 2% worldwide (Arvilommi et al. 2022; McIntyre et al. 2022). The disorder often presents in late adolescence or early adulthood and follows a relapsing‐remitting course that imposes substantial individual and societal burdens (Volkmann et al. 2020).

Despite advances in psychopharmacology and psychotherapy, the underlying neurobiological mechanisms of BD remain only partially understood. In recent years, there has been growing interest in elucidating the neuroanatomical and neurofunctional substrates of BD to better inform diagnosis, prognosis, and treatment strategies. A leading hypothesis posits that BD involves disrupted neural connectivity, particularly within circuits mediating emotional regulation, executive functioning, and cognitive control (Liberati et al. 2025). While early neuroimaging studies predominantly focused on gray matter abnormalities, emerging evidence from diffusion tensor imaging (DTI) studies has shifted attention toward white matter (WM) microstructure as a critical contributor to BD pathophysiology (Abi‐Dargham et al. 2023).

DTI enables in vivo assessment of WM integrity by measuring the directionality of water diffusion within axonal fibers, producing quantitative metrics such as fractional anisotropy (FA), mean diffusivity (MD), radial diffusivity (RD), and axial diffusivity (AD). Reduced FA and increased MD are frequently interpreted as indices of compromised WM integrity, reflecting demyelination, axonal loss, or other microstructural disruptions (Bergamino et al. 2021). Numerous DTI studies have reported WM abnormalities in individuals with BD, with frequently implicated tracts including the corpus callosum, cingulum bundle, uncinate fasciculus, and anterior thalamic radiation (ATR) (Madden et al. 2012). These tracts play crucial roles in mediating interhemispheric communication and linking prefrontal, limbic, and subcortical regions involved in mood regulation and cognitive processing.

However, the literature on WM alterations in BD remains inconsistent. Some studies have failed to detect significant differences in WM integrity between BD patients and healthy controls, while others have reported contradictory findings, such as increased FA in specific regions (Ren et al. 2020). These discrepancies may be attributed to variations in sample characteristics (BD subtype, illness duration, mood state), medication status, imaging acquisition protocols, and analytic methodologies. In addition, the relatively small sample sizes of many individual studies limit statistical power and generalizability.

Previous meta‐analyses have attempted to address these inconsistencies by aggregating findings across studies. Mahon et al. (2010) and Abramovic et al. (2018) provided early evidence of widespread WM disruptions in BD, particularly in fronto‐limbic and interhemispheric tracts (Mahon et al. 2013; Abramovic et al. 2018). Nonetheless, these analyses were limited by the number of included studies and the absence of spatial meta‐analytic techniques capable of identifying regions of consistent WM alterations across studies.

To address these limitations, the present study conducted a comprehensive systematic review and meta‐analysis of DTI studies examining WM tract alterations in BD. We compiled data from 145 studies, including over 11,000 participants, offering a wide overview of the currently available DTI literature in BD. In addition to standard meta‐analytic techniques evaluating group differences in FA, we employed exploratory spatial clustering of reported coordinates to identify spatial convergence in WM abnormalities and performed voxel count analyses to assess the extent of WM involvement across studies.

The objectives of this study were threefold. First, we aimed to quantify the magnitude of FA differences between individuals with BD and healthy controls across major WM tracts. Second, we sought to identify spatial patterns of WM alterations using exploratory spatial clustering of reported coordinates. Third, we aimed to evaluate the extent of WM disruptions by analyzing voxel count data. By synthesizing findings from a large and diverse body of research, this study provides an updated descriptive overview of WM microstructural findings in BD.

## 2 Methods

### 2.1 Study Design

This study is a systematic review and meta‐analysis conducted in accordance with the PRISMA guidelines (Page et al. 2021). The study protocol was registered in the Open Science Framework (OSF) (DOI 10.17605/OSF.IO/5AK7S).

### 2.2 Search Strategy

A comprehensive literature search was performed across the Web of Science, PubMed, and Scopus databases to identify relevant studies examining WM alterations in individuals with BD using DTI. The search was conducted in August 2024. Detailed search strategies for each database are provided in Table 1.

**TABLE 1:** Curated search strategies for each database.

Database	Search strategy	Results
Pubmed	(“Bipolar Disorder”[Title/Abstract] OR “Bipolar Disorder”[MeSH Terms] OR “Manic Disorders”[Title/Abstract] OR “Manic‐Depressive”[Title/Abstract] OR “Manic‐Depressive Psychosis”[Title/Abstract] OR “Bipolar Mood Disorders”[Title/Abstract] OR “Manic Depression”[Title/Abstract] OR “Bipolar Disorder Type 2”[Title/Abstract] OR “Type 2 Bipolar Disorder”[Title/Abstract]) AND (“Diffusion Tensor Imaging”[Title/Abstract] OR “Diffusion Tensor Imaging”[MeSH Terms] OR “Diffusion Tensor Magnetic Resonance Imaging”[Title/Abstract] OR “Diffusion Tensor MRI”[Title/Abstract] OR “Diffusion Tensor MRIs”[Title/Abstract] OR “Diffusion Tractography”[Title/Abstract]) AND (“White Matter”[Title/Abstract] OR “White Matter”[MeSH Terms] OR “White matter tract”[Title/Abstract] OR “White matter change”[Title/Abstract] OR “White matter alteration”[Title/Abstract])	293
WOS	((TS = (“white matter”) OR TS = (“white matter tract”)) OR TS = (“white matter change”)) OR TS = (“white matter alteration”)) AND ((((((TS = (“Bipolar Disorder”[) OR TS = (“Manic Disorders”)) OR TS = (“Manic‐Depressive”)) OR TS = (“Manic‐Depressive Psychosis”)) OR TS = (“Bipolar Mood Disorders”)) OR TS = (“Manic Depression”)) OR TS = (“Bipolar Disorder Type 2”)) OR TS = (“Type 2 Bipolar Disorder”)) AND (((TS = (“Diffusion Tensor Imaging”) OR TS = (“Diffusion Tensor Magnetic Resonance Imaging”)) OR TS = (“Diffusion Tensor MRI”)) OR TS = (“Diffusion Tensor MRIs”)) OR TS = (“Diffusion Tractography”))	394
Scopus	(TITLE‐ABS‐KEY(“Bipolar Disorder”) OR TITLE‐ABS‐KEY(“Manic Disorders”) OR TITLE‐ABS‐KEY(“Manic‐Depressive”) OR TITLE‐ABS‐KEY(“Manic‐Depressive Psychosis”) OR TITLE‐ABS‐KEY(“Bipolar Mood Disorders”) OR TITLE‐ABS‐KEY(“Manic Depression”) OR TITLE‐ABS‐KEY(“Bipolar Disorder Type 2”) OR TITLE‐ABS‐KEY(“Type 2 Bipolar Disorder”)) AND (TITLE‐ABS‐KEY(“white matter”) OR TITLE‐ABS‐KEY(“white matter tract”) OR TITLE‐ABS‐KEY(“white matter change”) OR TITLE‐ABS‐KEY(“white matter alteration”)) AND (TITLE‐ABS‐KEY(“Diffusion Tensor Imaging”) OR TITLE‐ABS‐KEY(“Diffusion Tensor Magnetic Resonance Imaging”) OR TITLE‐ABS‐KEY(“Diffusion Tensor MRI”) OR TITLE‐ABS‐KEY(“Diffusion Tensor MRIs”) OR TITLE‐ABS‐KEY(“Diffusion Tractography”))	409

### 2.3 Eligibility Criteria

Inclusion criteria were as follows:

We included studies enrolling participants with a clinical diagnosis of BD based on DSM or ICD criteria, irrespective of subtype (BD‐I or BD‐II). When subtype information was explicitly provided in the original articles, it was recorded during data extraction. Peer‐reviewed original studies investigating imaging biomarkers in individuals diagnosed with BD. Studies employing DTI to assess WM alterations. Human studies published in English.

Exclusion criteria were as follows:

Nonoriginal research (reviews, commentaries). Conference abstracts, case reports, and case series. Animal studies or those not involving human participants. Studies not focused on WM alterations in BD.

### 2.4 Study Selection and Data Extraction

Screening and selection of studies were performed independently by two reviewers using Rayyan software. Following screening, 145 unique studies met the eligibility criteria for the systematic review. Twenty‐eight studies provided extractable numerical FA data suitable for quantitative synthesis. These yielded 78 independent region‐level effect‐size entries, each corresponding to a distinct tract or subtract FA comparison.

Discrepancies were resolved by consensus with a third reviewer (M.A.A.). A structured data extraction sheet was used to ensure consistency, focusing on study demographics, participant characteristics, DTI metrics (e.g., FA), and reported MNI coordinates.

### 2.5 Quality Assessment

Two reviewers independently assessed the methodological quality of each study using the Newcastle‒Ottawa Scale. Any disagreements were resolved through discussion.

### 2.6 Spatial Data Analysis and Exploratory Spatial Clustering of Reported Coordinates

#### 2.6.1 Data Preparation

The coordinate dataset was treated as a descriptive spatial compilation rather than a formal input set for coordinate‐based meta‐analysis. Studies varied in how spatial findings were reported: some provided peak MNI coordinates directly, whereas others reported only anatomical labels. For the latter, atlas‐derived reference coordinates were assigned using the JHU ICBM‐DTI‐81 White Matter Labels Atlas solely to enable approximate spatial visualization. Because atlas‐imputed coordinates are not equivalent to reported peak effects, the resulting coordinate set should be interpreted with caution.

#### 2.6.2 Visualization of Spatial Data

A three‐dimensional (3D) spatial distribution of the cleaned MNI coordinates was visualized using Python's matplotlib library. Coordinates were plotted in MNI space, with axes representing the left‐right (*X*), anterior–posterior (*Y*), and inferior–superior (*Z*) directions. This visualization provided an overview of the distribution of reported WM alterations.

#### 2.6.3 Cluster Analysis

To investigate spatial convergence among reported coordinates, a *k*‐means clustering analysis was performed using scikit‐learn in Python. The optimal number of clusters was determined by evaluating silhouette scores across a range of cluster solutions (2–10 clusters). The highest silhouette score (0.81) indicated that a two‐cluster solution was optimal.

##### 2.6.3.1 Clustering Procedure

*k*‐Means clustering was applied to the dataset, grouping MNI coordinates based on their spatial proximity.

Cluster centroids (mean MNI *X*, *Y*, *Z*) and the number of points within each cluster were computed and reported.

A 3D scatter plot was generated, showing clusters with unique color codes and marking centroids with black “X” symbols.

### 2.7 Software and Tools

The following software and libraries were used for spatial data processing and analysis:

Python 3.x
pandas for data manipulation
numpy for numerical processing
matplotlib for 3D plotting
scikit‐learn for clustering analysis and silhouette score calculation

### 2.8 Statistical Analysis

All statistical analyses were conducted to quantitatively synthesize findings from DTI studies examining WM alterations in individuals with BD. The statistical strategy was developed following established guidelines for neuroimaging meta‐analyses and was informed by previous reviews and meta‐analyses (e.g., Mahon et al. 2010; Abramovic et al. 2018). Analyses were performed using Python (v3.x), Stata (v17), and specialized neuroimaging meta‐analysis tools.

For studies reporting group comparisons of FA between BD patients and healthy controls, random‐effects meta‐analyses were conducted to calculate pooled effect sizes (Hedges' *g*). This approach was chosen to account for heterogeneity in study design and participant characteristics. Standardized mean differences (SMDs) and their 95% confidence intervals (CIs) were calculated for each included study. The DerSimonian and Laird method was applied to estimate between‐study variance (*τ* ^2^).

Heterogeneity across studies was assessed using Cochran's *Q* test and quantified with the *I* ^2^ statistic. *I* ^2^ values greater than 75% were considered to indicate substantial heterogeneity. Subgroup analyses were performed to explore differences in FA reductions across distinct WM tracts (e.g., corpus callosum, uncinate fasciculus, cingulum bundle, ATR). The test of subgroup differences was evaluated with the Q statistic for between‐group heterogeneity.

An exploratory spatial clustering of reported coordinates was performed to identify regions of consistent WM alterations reported in MNI space. Studies that reported peak MNI coordinates of significant FA reductions were included in the analysis. Coordinates were pooled and analyzed using *k*‐means clustering (implemented in scikit‐learn v1.0, Python) to identify spatial convergence of reported abnormalities.

The optimal number of clusters was determined using silhouette score optimization across a range of two to ten clusters. Spatial distributions of MNI coordinates were visualized in 3D scatter plots. Cluster centroids and the number of contributing coordinates were calculated for each cluster. Regions were interpreted with reference to established WM atlases, including the JHU ICBM‐DTI‐81 White Matter Labels Atlas.

For studies reporting voxel counts of significant WM alterations, descriptive and meta‐analytic statistics were calculated. Voxel counts were analyzed by region, and the mean, standard deviation (SD), and 95% CIs were computed for each WM tract. Forest plots were generated to visualize regional voxel counts and their variability across studies. Due to the wide range of voxel counts, data were plotted on a logarithmic scale to facilitate interpretation of regional differences.

Publication bias was assessed through visual inspection of funnel plots and formally tested using Egger's regression asymmetry test. Funnel plot symmetry was evaluated for the overall meta‐analysis of FA differences and within key subgroup analyses. Egger's test *p* < 0.05 was considered indicative of potential publication bias.

Between‐study heterogeneity was further explored through Galbraith plots, which visually display standardized effect sizes against study precision to detect outliers contributing to heterogeneity. Formal leave‐one‐out influence diagnostics were not available for all subgroup models and, therefore, were not presented separately; the robustness of the findings was instead assessed through Galbraith‐plot inspection and exploratory outlier exclusion.

When reported in the original studies, acquisition characteristics such as scanner vendor, field strength, number of diffusion directions, *b* values, voxel dimensions, slice thickness, and analytic pipeline were reviewed qualitatively to aid interpretation of between‐study heterogeneity; however, reporting was too inconsistent to support a harmonized quantitative moderator analysis across all parameters.

### 2.9 Statistical Software

Data processing and statistical analyses were performed using:

Python (v3.x): Data cleaning, coordinate processing, *k*‐means clustering, and visualization (matplotlib and seaborn).

Stata (v17): Meta‐analysis of FA values, heterogeneity statistics, Egger's test, and forest/funnel plot generation.

Microsoft Excel (v365): Supplementary data preparation and verification.

ACE Tools (custom Python library): For interactive visualization of meta‐analytic datasets.

All statistical tests were two‐tailed, and significance was set at *p* < 0.05 unless otherwise specified. Because the included studies differ in methodology, acquisition parameters, cohort overlap likelihood, and reporting formats, the pooled estimates should be interpreted as reflecting cross‐study patterns rather than definitive population‐level effect magnitudes.

## 3 Results

### 3.1 Study Selection and Characteristics

**FIGURE 1:**
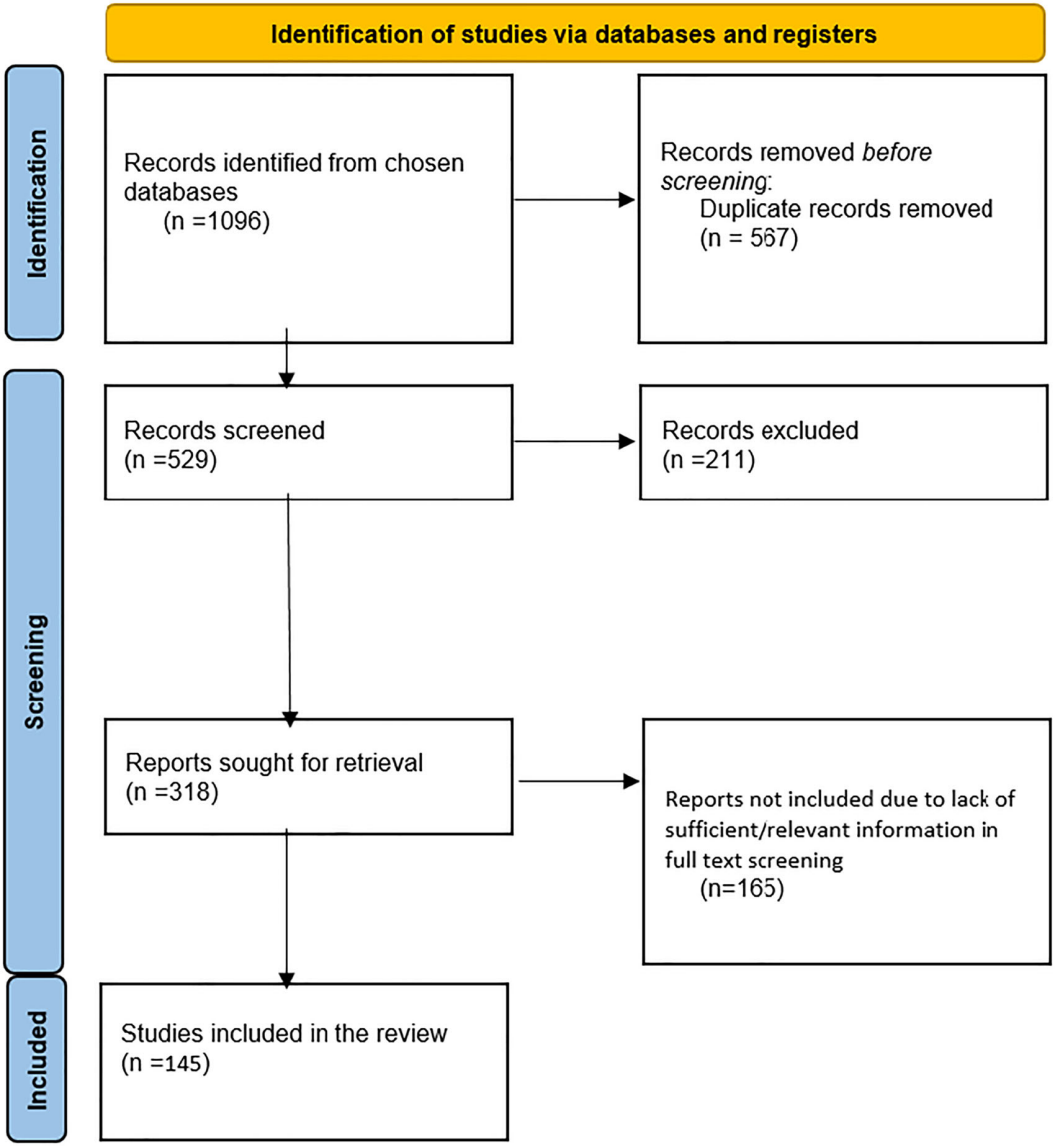
Prisma flow chart of the study selection procedure.

A total of 145 studies met the inclusion criteria for the systematic review, and 28 of these provided extractable tract‐level FA data for quantitative synthesis (Mahon et al. 2013; Adler et al. 2004, 2006; Aggio et al. 2023; Aghajani et al. 2014; Ajilore et al. 2015; Armstrong et al. 2020; Baumann et al. 2016; Benedetti et al. 2011, 2013, 2014, 2016, 2018; Berkovitch et al. 2021; Besenek et al. 2019; Besga et al. 2012, 2016; Beyer et al. 2005; Bollettini et al. 2015; Caseras et al. 2015; Cha et al. 2022; Chan et al. 2010; Chou et al. 2014; Cruz‐Sanabria et al. 2021; Cui et al. 2011; Cyprien et al. 2016; Deng et al. 2018; Fan et al. 2019; Fernandez‐Cabello et al. 2022; Foley et al. 2018; Gao et al. 2013; Goghari et al. 2021; Gönenç et al. 2010; Guo et al. 2021; Haarman et al. 2016; Haller et al. 2011; Hanlon et al. 2021; Hatano et al. 2019; Hawco et al. 2017; Hermens et al. 2018, 2019; Ho et al. 2017; Hou et al. 2022; Houenou et al. 2007; Ishida et al. 2017; Ji et al. 2019; Jiang et al. 2022, 2023; Kafantaris et al. 2017; Koreki et al. 2022; Kurumaji et al. 2017; Kuswanto et al. 2013, 2014; Lan et al. 2020; Lee et al. 2020, 2022; Leow et al. 2013; J. Li et al. 2014; X. Li et al. 2021; Lin et al. 2011; Linke et al. 2013; Liu et al. 2010; Maeno et al. 2007; Mahapatra et al. 2017; E. Mallas et al. 2017; E.‐J. Mallas et al. 2016; Martino et al. 2016; Masuda et al. 2020; Mazza et al. 2017; McKenna et al. 2015; Melloni et al. 2019; Nabulsi et al. 2023; Nazeri et al. 2017; Nenadić et al. 2017; Niida et al. 2018; O'Donoghue et al. 2017; Oertel‐Knöchel et al. 2014; Ota et al. 2016; Pan et al. 2022; Pavuluri et al. 2009; Piaggio et al. 2018; Poletti, Bollettini, et al. 2015; Poletti, Mazza, et al. 2015; Price et al. 2007; Reckziegel et al. 2022; Ren et al. 2020; Roberts et al. 2016, 2022; Romero et al. 2022; Saglam et al. 2023; Sariçiçek et al. 2016; Shahab et al. 2019; Singh et al. 2023; Sivagnanasundaram et al. 2007; Skudlarski et al. 2013; Sprooten et al. 2012, 2016; Stein et al. 2022; Stevelink et al. 2018; Sui et al. 2011; Sun et al. 2024; F. Tang et al. 2020; Y. Tang et al. 2018; Tønnesen et al. 2020; Torgerson et al. 2013; Toteja et al. 2015; Vandekerckhove et al. 2020; Verkooijen et al. 2017; Versace et al. 2008, 2014, 2018; Y. Wang et al. 2019; F. Wang et al. 2008, 2009; B. Wang et al. 2018; Whalley et al. 2015; White et al. 2015; Winterer et al. 2008; R. Zhang et al. 2019; S. Zhang et al. 2018; Zhou et al. 2022). Across the 145 included studies, a total of 5372 individuals with BD and 6240 healthy controls were identified. These values reflect study‐level deduplication, where each cohort contributed participant data only once, even if multiple brain regions or analytic contrasts were reported. The mean age of the BD group was 35.9 years (SD = 0.8), while the mean age of the control group was 34.8 years (SD = 0.7). All included studies used DTI to assess WM microstructure, primarily through FA measures (Figure 1).

The pooled FA analyses reported below are based only on the 28 studies that contributed extractable quantitative data.

### 3.2 Quantitative Meta‐Analysis of FA

The overall meta‐analysis using a random‐effects model demonstrated a significant reduction in FA among individuals with BD compared to healthy controls:

Hedges' *g* = −0.56 (95% CI: −0.71 to −0.42; *p* < 0.001).

**FIGURE 2:**
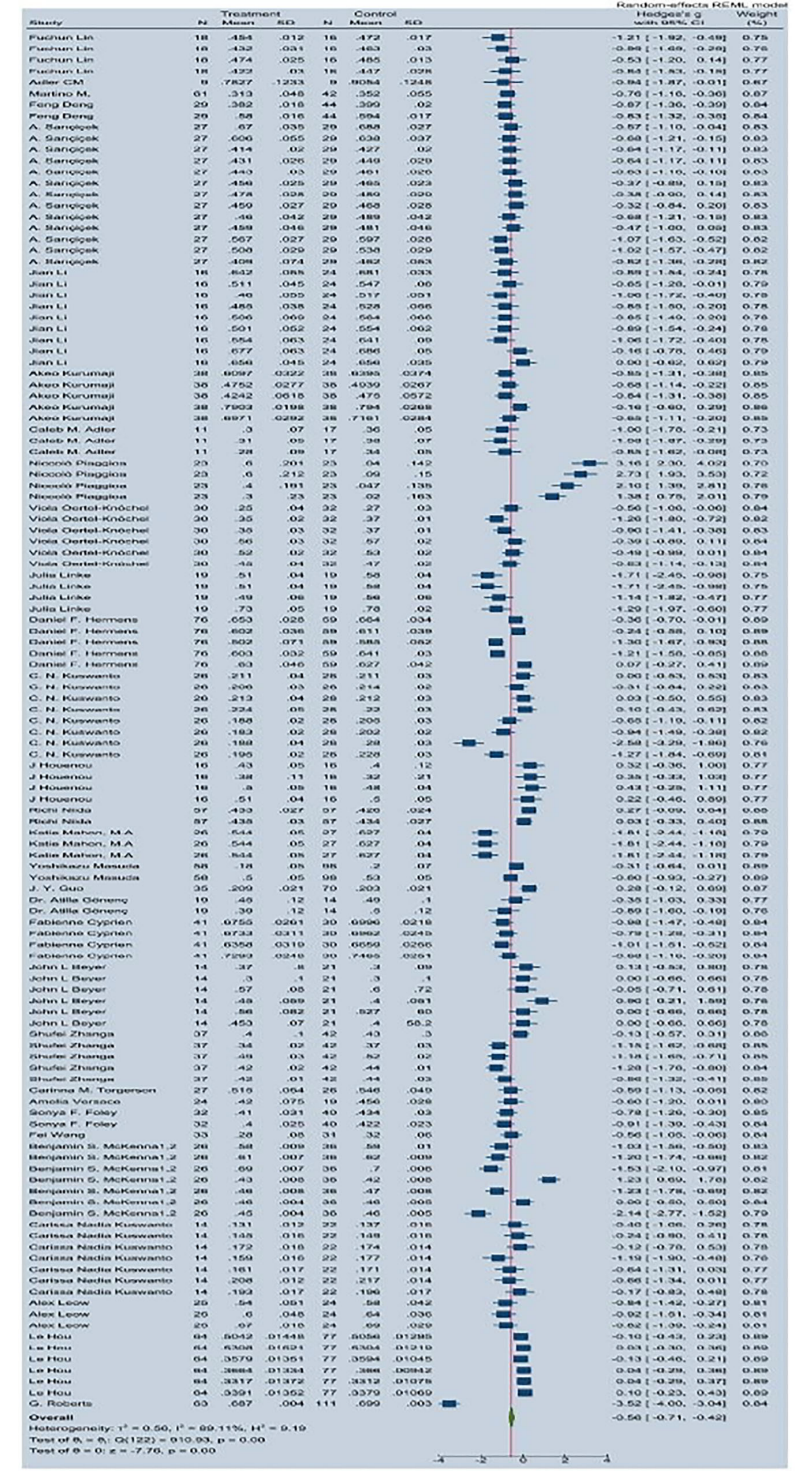
Forest plot of FA differences (Hedges' *g*) between BD patients and controls.

The between‐study heterogeneity was substantial (*I* ^2^ = 89.11%), indicating considerable variation across studies (Figure 2).

### 3.3 Publication Bias and Heterogeneity

**FIGURE 3:**
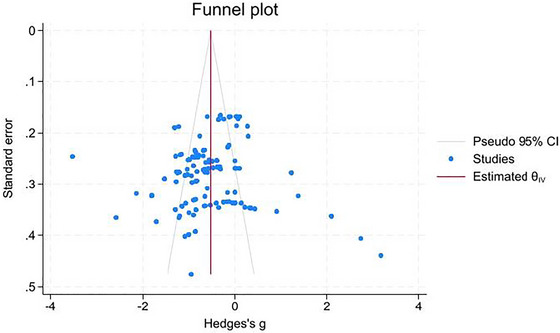
Funnel plot assessing publication bias.

**FIGURE 4:**
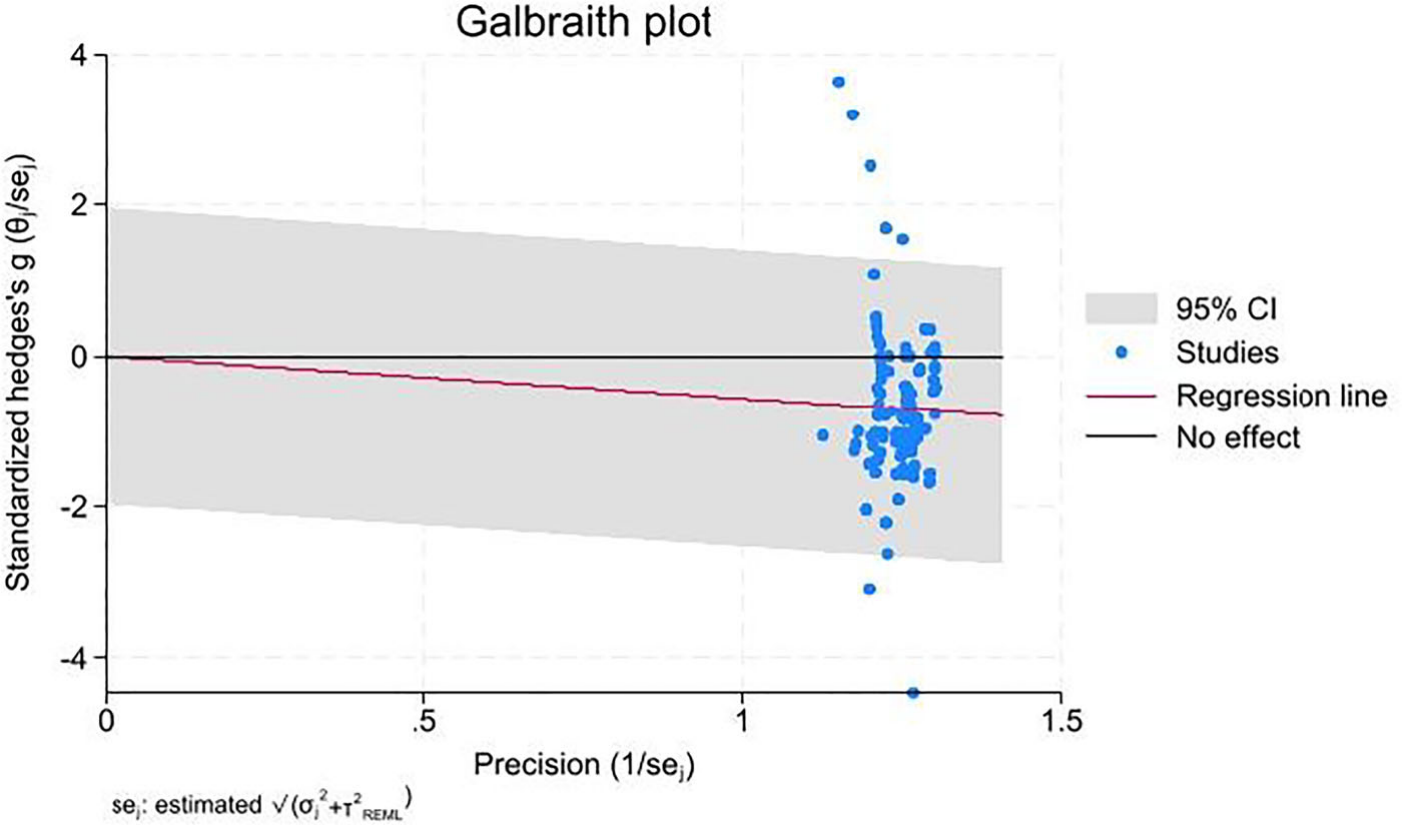
Galbraith plot assessing study heterogeneity.

Egger's regression test revealed no significant evidence of publication bias (*p* > 0.05). The funnel plot showed symmetry around the average effect size, confirming the absence of publication bias (Figure 3). The Galbraith plot highlighted heterogeneity, with several studies lying outside the 95% confidence limits (Figure 4).

### 3.4 Subgroup Meta‐Analysis by Altered Brain Regions

A subgroup meta‐analysis was conducted based on specific WM tracts and regions implicated in BD. The results demonstrated region‐specific variations in the magnitude of FA reductions (Table 2).

**TABLE 2:** Subgroup meta‐analysis of white matter tract fractional anisotropy alterations in bipolar disorder.

White‐matter region/tract	Pooled effect (Hedges' *g*)	95% CI	*p*
Anterior thalamic radiation	−0.27	[−0.51, −0.03]	< 0.05
Cingulum bundle	−0.64	[−1.15, −0.13]	< 0.05
Corona radiata (anterior)	−0.46	[−0.82, −0.10]	< 0.05
Corona radiata (posterior)	−0.48	[−0.86, −0.09]	< 0.05
Corona radiata (superior)	−0.63	[−1.09, −0.18]	< 0.01
Corpus callosum (body)	−0.67	[−0.91, −0.43]	< 0.001
Corpus callosum (splenium)	−0.84	[−1.29, −0.39]	< 0.001
Corpus callosum (whole)	−3.54	[−6.23, −0.84]	< 0.01
Corticospinal tract	−0.86	[−1.24, −0.49]	< 0.001
External capsule	−0.91	[−1.24, −0.58]	< 0.001
Superior longitudinal fasciculus	−0.53	[−1.02, −0.03]	< 0.05
Uncinate fasciculus	−0.66	[−0.91, −0.42]	< 0.001
Overall	−0.56	[−0.71, −0.42]	< 0.001

*Note*: Tract‐specific pooled effect sizes are shown as Hedges' *g* with 95% confidence intervals and *p* values from random‐effects models. Negative effect sizes indicate reduced fractional anisotropy in bipolar disorder relative to healthy controls.

ATR: Small but significant FA reduction

Hedges' *g* = −0.27 (95% CI: −0.51 to −0.03; *p* < 0.05)

Cingulum bundle: Moderate reduction in FA

Hedges' *g* = −0.64 (95% CI: −1.15 to −0.13; *p* < 0.05)

Corona radiata

Anterior: *g* = −0.46 (95% CI: −0.82 to −0.10; *p* < 0.05)

Posterior: *g* = −0.48 (95% CI: −0.86 to −0.09; *p* < 0.05)

Superior: *g* = −0.63 (95% CI: −1.09 to −0.18; *p* < 0.01)

Corpus callosum

Body: *g* = −0.67 (95% CI: −0.91 to −0.43; *p* < 0.001)

Splenium: *g* = −0.84 (95% CI: −1.29 to −0.39; *p* < 0.001)

Whole: *g* = −3.54 (95% CI: −6.23 to −0.84; *p* < 0.01)

The estimate for the whole corpus callosum was notably larger in magnitude than the other tract‐level effects and should therefore be interpreted cautiously. This unusually large value may reflect a small number of contributing entries, differences in regional definition, or scaling and reporting variation in source FA values rather than a stable tract‐wide effect of this magnitude.

Corticospinal tract: *g* = −0.86 (95% CI: −1.24 to −0.49; *p* < 0.001)

External capsule: *g* = −0.91 (95% CI: −1.24 to −0.58; *p* < 0.001)

Superior longitudinal fasciculus: *g* = −0.53 (95% CI: −1.02 to −0.03; *p* < 0.05)

Uncinate fasciculus: *g* = −0.66 (95% CI: −0.91 to −0.42; *p* < 0.001)

### 3.5 Subgroup Differences

The test for subgroup differences was significant (*Q* = 105.39; *p* < 0.001), indicating that the degree of FA reduction varied across different WM tracts.

#### 3.5.1 “Unclassified” and “Other Brain Region” Categories

This category included brain regions that were either:

Nonspecific,
Reported as nonsignificant between the BD and control groups,
Or anatomically ambiguous.
Examples include
Left ATR
Corpus callosum (nonspecific region)
Forceps major
Posterior thalamic radiation (L)
Sagittal stratum (L)
Precuneus (R)
Isthmus
Truncus corpus callosum
Anterior limb of the internal capsule
Retrolenticular part of the internal capsule
Pons–cerebellum connections
SC–AH tracts (bilateral)
Multiple general anatomical terms

“Other brain regions”

This category consisted of diverse regions that did not fit into standard WM tracts but were consistently reported as altered.

Examples include:

Prefrontal WM at multiple levels superior to the AC
Superior fronto‐occipital fasciculus (L)
Frontal–parietal lobes (L), frontal lobes (R), occipital lobes (L and R)
Precuneus (L)
Anterior cingulate cortex (L and R)
Temporal WM regions
Orbitofrontal cortex
Pars opercularis, insula
Cortico‐ponto‐cerebellar tracts
Cerebellar tracts (superior, middle, inferior)

These regions were grouped under “other brain regions” to capture the breadth of findings beyond classic WM tracts.

### 3.6 Exploratory Spatial Clustering of Reported Coordinates

**FIGURE 5:**
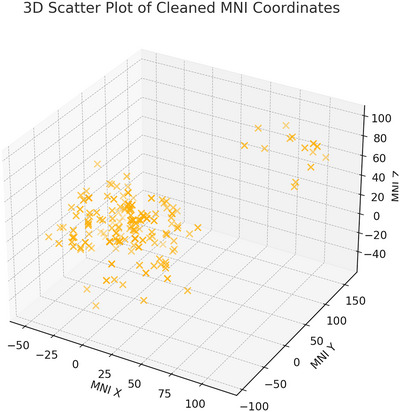
3D scatter plot of cleaned MNI coordinates.

An exploratory spatial clustering of reported coordinates was conducted using 225 valid MNI coordinates extracted from the dataset:

A 3D scatter plot illustrates the spatial distribution of reported WM alterations (Figure 5).

**FIGURE 6:**
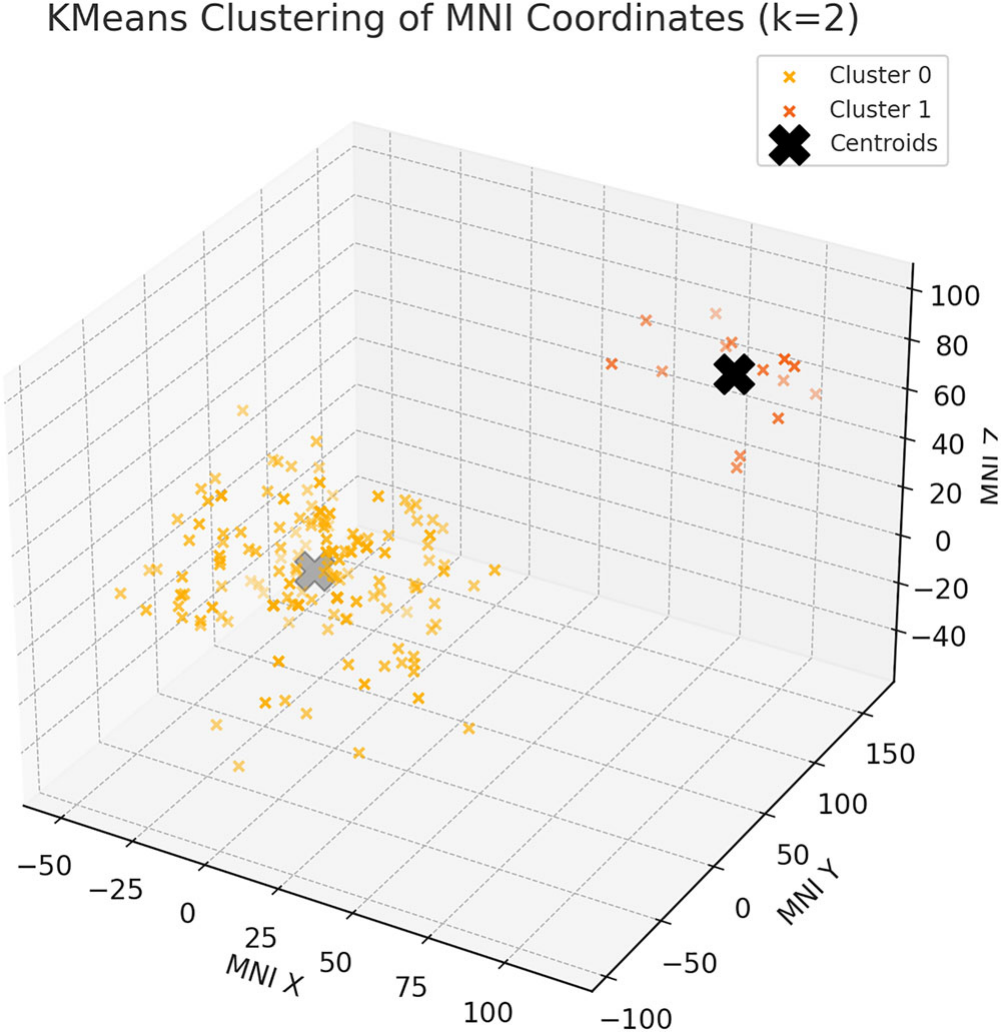
*k*‐Means clustering of MNI coordinates (*k* = 2).

*k*‐Means clustering revealed two distinct spatial clusters (Figure 6):

Cluster 1: Centered at MNI (−3.00, −8.13, 13.97); 210 points, reflecting midline structures (e.g., corpus callosum, cingulum bundle).

Cluster 2: Centered at MNI (90.53, 121.87, 75.87); 15 points, reflecting lateralized or less frequently reported regions.

An exploratory spatial clustering analysis was conducted using 225 valid MNI coordinates extracted from the included studies. After cleaning and standardizing the coordinate data, a 3D scatter plot was generated to visualize the spatial distribution of reported WM abnormalities across MNI space (Figure 5). The scatter plot showed a heterogeneous distribution of coordinates with visible concentration in midline WM regions.

*k*‐Means clustering identified a two‐cluster solution as optimal (silhouette score = 0.81), indicating a strong clustering structure (Figure 6). Cluster 1 was centered at MNI (−3.00, −8.13, 13.97) and included 210 coordinate points, suggesting convergence in midline WM tracts such as the corpus callosum and cingulum bundle. Cluster 2 was centered at MNI (90.53, 121.87, 75.87) and included 15 coordinate points, representing a smaller and more lateralized distribution that may reflect less commonly reported or outlying regions.

These spatial summaries should be interpreted descriptively rather than inferentially. The coordinate set combined directly reported MNI peaks with atlas‐derived reference coordinates for label‐only reports, and a small subset of coordinates occupied extreme positions after standardization. Accordingly, this clustering analysis is best understood as a visualization of how reported abnormalities were distributed across the literature rather than as a formal coordinate‐based meta‐analysis such as ALE or SDM‐PSI.

### 3.7 Meta‐Analysis of Voxel Counts by Region

In addition to analyzing FA differences, a separate meta‐analysis was conducted to explore the extent of WM involvement by examining the voxel counts reported across included studies. Voxel counts serve as an indicator of the spatial extent (volume) of reported WM alterations in specific regions, offering a complementary measure to effect sizes such as FA reductions. The aim was to determine whether certain regions consistently demonstrated larger spatial involvement in individuals with BD.

### 3.8 Data Overview

Voxel count data were available for a subset of the studies, with 183 entries for voxels_cleaned, 138 entries for voxel_count_cleaned, and 45 entries for voxel_size_cleaned. The meta‐analysis focused on regions where voxel count data were sufficiently reported to allow for summary statistics and meaningful comparisons.

### 3.9 Analytical Approach

Voxel counts were aggregated by WM region, as indicated in the abr_cleaned column. For each region, the mean voxel count, SD, and number of studies contributing data were calculated. These descriptive statistics provided insights into the average spatial extent of reported abnormalities and the variability across studies.

**FIGURE 7:**
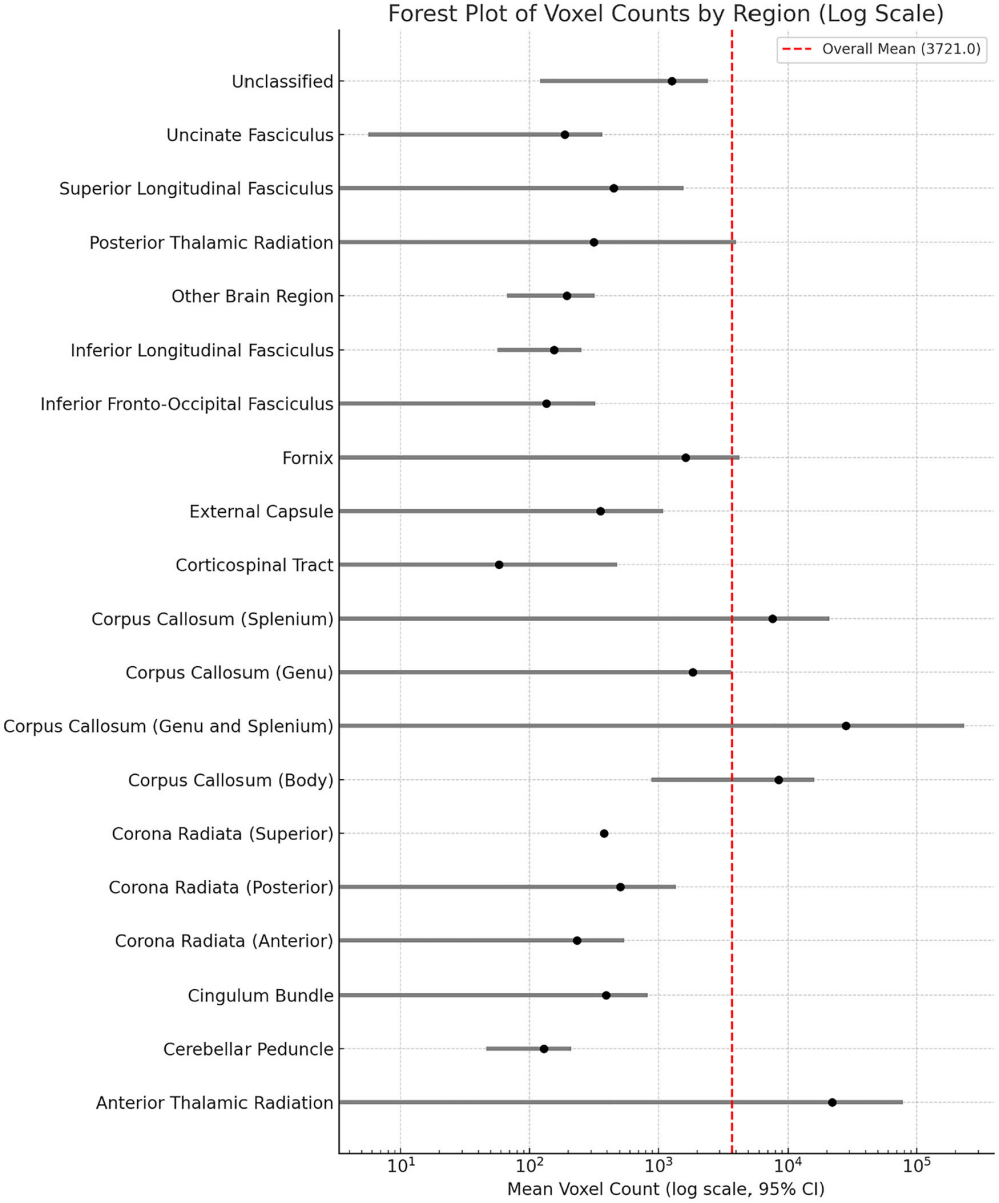
Forest plot of voxel counts by region (log scale).

A forest plot was constructed to display the mean voxel counts and their 95% CIs for each WM region (Figure 7). Due to the wide range of voxel counts across regions, the data were plotted on a logarithmic scale to facilitate visual comparison.

ATR exhibited the highest mean voxel count, with an average of 21,994 voxels (SD = 44,902). This finding suggests that alterations in the ATR may extend over large volumes of WM, potentially reflecting widespread disruption of thalamocortical connectivity in BD.

The corpus callosum (body) was the most frequently studied region in terms of voxel counts, with 14 contributing studies and an average voxel count of 8462 voxels (SD = 13,123). This substantial involvement highlights the importance of interhemispheric communication deficits in BD.

The corpus callosum (splenium) and corona radiata (posterior) also demonstrated notable spatial involvement, with mean voxel counts of 7558 voxels and 504 voxels, respectively. These regions are involved in sensory‐motor integration and cognitive processing, suggesting their potential role in BD pathophysiology.

Regions such as the cingulum bundle and corona radiata (anterior) showed relatively lower voxel counts, averaging 392 voxels and 233 voxels, respectively. While these values indicate smaller spatial extents of reported abnormalities, their consistent inclusion across studies and significant FA reductions underscore their relevance in BD.

Regions with single study contributions, such as the corona radiata (superior) and corpus callosum (whole), demonstrated high variability or lacked measures of dispersion due to limited data. Their inclusion provides completeness but requires cautious interpretation.

### 3.10 Interpretation

Voxel count results should be interpreted as relative indicators of the spatial extent of reported abnormalities rather than absolute volumetric measures. Direct comparison across studies is limited by differences in voxel size, slice thickness, preprocessing workflow, and statistical thresholding strategy, including FWE‐corrected, FDR‐corrected, TFCE‐based, and uncorrected analyses. In addition, some studies reported cluster extent in voxels, whereas others reported values in mm^3^. These factors mean that the voxel‐count analysis is most useful for descriptive contextualization of regional involvement rather than precise between‐study volume quantification.

## 4 Discussion

This study combined a broad systematic review with a narrower quantitative synthesis to characterize WM abnormalities in BD. The 145‐study systematic review provided a comprehensive overview of the DTI literature and supported descriptive analyses of reported spatial patterns and regional involvement. Within this broader evidence base, the 28‐study quantitative synthesis demonstrated significant reductions in FA across multiple WM tracts, including the corpus callosum, cingulum bundle, uncinate fasciculus, ATR, and corona radiata. Together, these findings suggest distributed but anatomically selective disruption of WM pathways involved in emotional regulation, cognitive control, and interhemispheric communication in BD (Favre et al. 2019).

Our findings are consistent with earlier meta‐analyses that reported compromised WM integrity in BD. Mahon et al. (2010) first identified FA reductions in the corpus callosum and cingulum bundle, regions implicated in emotional regulation and cognitive processing (Mahon et al. 2013). Abramovic et al. (2018) expanded on these findings, demonstrating widespread WM disruptions with a focus on tracts involved in mood regulation. Our findings align with and expand upon previous evidence by incorporating a broader range of published studies and participant samples than earlier reviews (Abramovic et al. 2018; Houenou et al. 2011).

Moreover, our voxel count analysis, which revealed the largest spatial extent of alterations in the ATR and corpus callosum, supports the hypothesis that thalamocortical dysconnectivity plays a central role in BD pathophysiology. However, there remains some inconsistency in the literature. For instance, Todd et al. observed elevated FA in the genu of the corpus callosum, a result not replicated in our study. Differences in patient selection, mood states at the time of scanning, medication use, and imaging methodologies likely account for these discrepancies (Yurgelun‐Todd et al. 2007).

Notably, our subgroup analysis highlighted tract‐specific variability in FA reductions. Significant alterations were identified in both anterior and posterior components of the corona radiata, as well as in the uncinate fasciculus, external capsule, and superior longitudinal fasciculus—regions less emphasized in earlier meta‐analyses. These findings reinforce the view that BD is associated with diffuse WM pathology extending beyond traditionally studied tracts (McCarthy‐Jones et al. 2018).

This exploratory spatial clustering of reported coordinates revealed two primary spatial clusters of WM abnormalities, with one cluster centered on midline structures (e.g., corpus callosum, cingulum) and a smaller cluster comprising more lateralized regions. These findings suggest a degree of anatomical consistency in reported abnormalities across studies despite methodological heterogeneity.

### 4.1 Clinical Implications

The pattern of WM microstructural abnormalities identified in this meta‐analysis carries potential clinical significance, particularly in the evolving field of neurobiological biomarkers for BD. The consistent FA reductions within the corpus callosum, cingulum bundle, uncinate fasciculus, ATR, and corona radiata suggest that BD may involve a trait‐level disruption of fronto‐limbic and thalamo‐cortical connectivity, circuits fundamental to emotional regulation, cognitive flexibility, and interhemispheric integration. From a biomarker perspective, these findings have relevance in several domains. First, the reproducibility of tract‐specific alterations across independent studies indicates potential utility as a diagnostic or risk‐stratification biomarker, particularly when considered in conjunction with genetic, inflammatory, or neurocognitive profiles. Second, the spatial distribution of abnormalities, especially within the uncinate fasciculus and cingulum, aligns with neural networks previously implicated in early‐onset, familial‐risk, and youth‐at‐risk cohorts, supporting investigation into endophenotype or vulnerability biomarkers. Third, longitudinal imaging studies suggest that WM abnormalities may evolve with illness duration and treatment exposure, raising the possibility that DTI measures could serve as progression or prognostic biomarkers, especially in differentiating neurodevelopmental trajectories from neuroprogressive changes. Fourth, emerging evidence linking tract‐specific integrity to medication response (e.g., lithium, mood stabilizers, and neuromodulation treatments such as repetitive TMS) highlights the exploratory potential for treatment‐response or stratification biomarkers, where pretreatment diffusion metrics might help predict who will benefit from specific interventions (Kafantaris et al. 2017; Bollettini et al. 2015; Shamabadi et al. 2023; Benedetti et al. 2018). Finally, given that DTI is sensitive but not disorder‐specific, future biomarker development will likely require multimodal integration (DTI + functional connectivity + polygenic risk + digital phenotyping), harmonized imaging protocols, and prospective validation within clinical decision‐making frameworks.

These findings, therefore, represent a meaningful step toward biomarker discovery in BD, but translation into clinical utility will require standardized acquisition methods, normative reference datasets, and prospective validation across illness stages and treatment settings.

### 4.2 Future Directions

While this meta‐analysis offers a comprehensive synthesis of existing DTI findings, several areas warrant further exploration. Longitudinal studies are necessary to clarify the temporal dynamics of WM alterations in BD, including whether these abnormalities represent trait markers or state‐dependent phenomena. In addition, multimodal imaging approaches integrating DTI with functional MRI (fMRI) and magnetic resonance spectroscopy (MRS) may provide a more holistic understanding of the interplay between WM structure, brain function, and neurochemistry (Konarski et al. 2006; Ogłodek et al. 2026).

The application of advanced diffusion imaging techniques, such as neurite orientation dispersion and density imaging (NODDI) and diffusion kurtosis imaging (DKI), could yield more nuanced insight into the microstructural properties of WM. Future research should also examine the effects of pharmacotherapy, illness duration, and clinical subtypes (BD‐I vs. BD‐II) on WM integrity. Moreover, machine learning and network‐based analyses may help develop individualized neuroimaging biomarkers for clinical use (Konarski et al. 2006; Ogłodek et al. 2026; Colombo et al. 2022). Complementary multimodal imaging approaches should also be incorporated in future work to bridge the gap between microstructural findings and functional network behavior. Integrating diffusion‐based measures with modalities such as resting‐state and task‐based fMRI, morphometric analyses, or MRS would allow researchers to relate tract‐level alterations to network connectivity, neural activity patterns, and neurochemical profiles. This combined framework could help determine whether WM abnormalities contribute to clinically relevant dysfunction at the systems level, ultimately improving mechanistic interpretation and biomarker development.

### 4.3 Strengths and Limitations

This meta‐analysis possesses several notable strengths that enhance the reliability, validity, and interpretability of its findings. One strength of this work is its wide inclusion of published datasets, with data extracted from 28 studies involving 11,612 participants. The inclusion of a broad and diverse set of published samples increases the descriptive breadth of findings, although underlying variability limits assumptions about population‐level generalizability.

A second key strength lies in the methodological rigor with which this study was conducted. The systematic review and meta‐analysis adhered strictly to PRISMA guidelines, ensuring transparency and reproducibility in study selection, data extraction, and quality assessment. Multiple advanced analytic techniques were employed to provide a multidimensional understanding of WM microstructure in BD. Specifically, the use of random‐effects models accounted for between‐study heterogeneity, while subgroup analyses offered insights into region‐specific alterations. Furthermore, the inclusion of exploratory spatial clustering of reported coordinates and voxel count analyses provided an additional spatial dimension to the interpretation of findings, identifying areas of convergence across studies and quantifying the extent of WM abnormalities.

An additional strength of this synthesis is that it provides a tract‐level structural framework that can be integrated with complementary biomarker modalities. WM abnormalities may prove most informative when interpreted alongside functional connectivity measures from fMRI, neurochemical indices from spectroscopy, structural MRI findings, genetic or polygenic risk measures, and cognitive or clinical markers. Such multimodal integration may help determine whether the observed alterations reflect disorder liability, illness progression, treatment‐related variation, or clinically meaningful biotypes.

In addition to methodological robustness, this study offers novel region‐specific insights that extend previous meta‐analyses. While prior studies have primarily focused on global measures of FA, the present analysis identified specific WM tracts most consistently affected in BD. Prominent among these were the corpus callosum, cingulum bundle, uncinate fasciculus, and ATR. The analysis also highlighted less frequently reported regions such as the external capsule and superior longitudinal fasciculus, underscoring the widespread and multifaceted nature of WM pathology in BD. Moreover, the absence of publication bias, confirmed through Egger's regression test, adds confidence to the reliability of these findings. Heterogeneity was systematically examined and visualized through Galbraith plots, offering transparency in how variability was addressed.

Despite these strengths, several limitations should be acknowledged. The most significant limitation of this meta‐analysis is the substantial heterogeneity observed across studies. The calculated *I* ^2^ statistic of 89.11% suggests considerable variability in the magnitude and direction of reported findings. This heterogeneity likely reflects differences in DTI acquisition protocols, analysis pipelines, participant characteristics (including age, sex, and illness duration), medication status, and mood states at the time of imaging. Although random‐effects models were employed to mitigate the impact of such variability, they remain an inherent limitation in synthesizing neuroimaging data from heterogeneous sources.

Part of the observed heterogeneity likely reflects cross‐platform and acquisition‐related variability rather than biological differences alone. Across the included literature, studies differed in scanner vendor, field strength, diffusion sampling scheme, *b*‐values, voxel dimensions, slice thickness, and analytic approach, including TBSS, tractography, and ROI‐based analyses. Each of these factors can influence FA estimates independently of diagnosis and may therefore have contributed to the dispersion of pooled effects. Accordingly, the pooled results should be interpreted as summary patterns across heterogeneous imaging platforms rather than platform‐invariant estimates of effect magnitude.

Another limitation is that most included studies employed cross‐sectional designs, limiting causal inferences regarding the progression of WM abnormalities over time. Longitudinal studies are essential to determine whether these abnormalities precede the onset of BD, progress with the illness course, or are influenced by pharmacological treatment. Furthermore, while the voxel count meta‐analysis provided valuable information on the spatial extent of WM alterations, the inconsistent reporting of voxel sizes across studies hindered the ability to convert these findings into absolute volumetric measurements. Standardization of voxel size reporting and the use of harmonized imaging pipelines are crucial for improving cross‐study comparability in future research.

Potential confounding factors such as the effects of psychotropic medications, comorbid psychiatric conditions, and mood state variability were not consistently controlled across studies. This lack of uniform reporting precluded the possibility of conducting subgroup analyses to account for these factors. In addition, while FA remains the most widely reported DTI metric, it offers limited specificity regarding the nature of WM microstructure abnormalities. Advanced diffusion imaging models, such as NODDI and DKI, may provide more nuanced insights into WM pathology in BD and should be incorporated into future studies.

Finally, although Egger's test suggested no significant publication bias, this meta‐analysis was limited to English‐language studies, which may introduce selection bias. The inclusion of non‐English publications in future systematic reviews could enhance the comprehensiveness and generalizability of findings.

While this meta‐analysis presents robust evidence of widespread WM alterations in BD, it is important to interpret these findings within the context of existing methodological limitations. Addressing these limitations in future longitudinal, multimodal imaging studies will be critical to advancing our understanding of the role of WM pathology in the etiology, course, and treatment of BD.

## 5 Conclusion

This meta‐analysis provides compelling evidence of widespread and regionally specific WM abnormalities in BD. Reductions in FA were most pronounced in the corpus callosum, cingulum bundle, uncinate fasciculus, and ATR, with spatial convergence highlighting midline and thalamocortical pathways. These findings underscore the role of disrupted structural connectivity in the pathophysiology of BD and have important implications for the development of neuroimaging biomarkers and targeted interventions. Addressing the methodological limitations identified in this study through longitudinal, multimodal, and standardized research approaches will be essential for advancing our understanding of WM pathology in BD.

## Author Contributions

**Milad Yousefi**: data curation, writing – review and editing, writing – original draft, visualization, validation, methodology. **Mohammad Javad Khosravi**: writing – original draft, validation, investigation. **Negarsadat Namazi**: writing – original draft, writing – review and editing. **Alisa Mohebbi**: writing – original draft, writing – review and editing. **Mahsa Asadi Anar**: writing – review and editing, investigation, writing – original draft, conceptualization, supervision, validation, visualization, methodology, project administration. **Behnaz Mahmoudvand**: writing – review and editing, writing – original draft, validation. **Zahra Rahimian**: investigation, writing – original draft, writing – review and editing. **Mohammad Saeed Soleimani Meigoli**: writing – review and editing, writing – original draft. **Mohammad Sadra Saghafi**: investigation, writing – original draft, writing – review and editing, formal analysis. **Hanna Torkzadeh**: writing – review and editing, writing – original draft, investigation. **Amirhossein Rigi**: conceptualization, writing – original draft, writing – review and editing. **Mina Jafari**: methodology, visualization, writing – review and editing, writing – original draft, formal analysis. **Hadi Mahdavian**: investigation, writing – original draft, writing – review and editing. **Mohaddeseh Sadat Hosseini**: data curation, writing – review and editing, writing – original draft. **Farbod Khosravi**: writing – review and editing, writing – original draft, investigation, validation. **Shaghayegh Karami**: validation, writing – review and editing, writing – original draft. **Melika Arab Bafrani**: investigation, writing – original draft, writing – review and editing. **Shadi Hajizamani**: writing – original draft, writing – review and editing, data curation. **Seyed Amirali Zakavi**: writing – review and editing, writing – original draft, conceptualization.

## Funding

The authors have nothing to report.

## Ethics Statement

The authors have nothing to report.

## Consent

The authors have nothing to report.

## Conflicts of Interest

The authors declare no conflicts of interest.

## Data Availability

The data are available upon reasonable request from the corresponding author.
